# Integrating cfDNA liquid biopsy and organoid-based drug screening reveals PI3K signaling as a promising therapeutic target in colorectal cancer

**DOI:** 10.1186/s12967-023-04675-6

**Published:** 2024-02-03

**Authors:** Huan Yang, Xing Xiao, Leli Zeng, Haiteng Zeng, Yueyuan Zheng, Jingshu Wang, Guanghua Li, Weigang Dai, Yulong He, Suihai Wang, Jianjun Peng, Wei Chen

**Affiliations:** 1 Department of Gastrointestinal Surgery, The First Affiliated Hospital of Sun Yat-Sen University, Guangzhou, Guangdong China; 2 Guangdong Provincial Key Laboratory of Digestive Cancer Research, Shenzhen, Guangdong China; 3 Department of Biobank, The Seventh Affiliated Hospital of Sun Yat-Sen University, Shenzhen, Guangdong China; 4 Clinical Big Data Research Center, Scientific Research Center, The Seventh Affiliated Hospital of Sun Yat-Sen University, Shenzhen, China; 5 Department of Oncology, Sun Yat-Sen Memorial Hospital, Sun Yat-Sen University, Guangzhou, Guangdong China; 6 Department of Gastrointestinal Surgery, The Seventh Affiliated Hospital of Sun Yat-Sen University, Shenzhen, Guangdong China; 7 School of Laboratory Medicine and Biotechnology, Southern Medical University, Guangzhou, Guangdong China

**Keywords:** Liquid biopsy, cfDNA, Colorectal cancer, Organoid, PIK3CA, Alpelisib

## Abstract

**Background:**

The current precision medicine relies on biomarkers, which are mainly obtained through next-generation sequencing (NGS). However, this model failed to find effective drugs for most cancer patients. This study tried to combine liquid biopsy with functional drug tests using organoid models to find potential drugs for cancer patients.

**Methods:**

Colorectal cancer (CRC) patients were prospectively enrolled and blood samples were collected from patients before the start of treatment. Targeted deep sequencing of cfDNA samples was performed using a 14-gene panel. Gastrointestinal (GI) cancer organoids were established and PI3K and mTOR inhibitors were evaluated on organoid models.

**Results:**

A total of 195 mutations were detected across 58 cfDNA samples. The most frequently mutated genes were KRAS, TP53, PIK3CA, and BRAF, all of which exhibited higher mutation rates than tissue biopsy. Although 81% of variants had an allele frequency of less than 1%, certain mutations in KRAS, TP53, and SMAD4 had high allele frequencies exceeding 10%. Notably, among the seven patients with high allele frequency mutations, six had metastatic tumors, indicating that a high allele frequency of ctDNA could potentially serve as a biomarker of later-stage cancer. A high rate of PIK3CA mutation (31 out of 67, or 46.3%) was discovered in CRC patients, suggesting possible tumor progression mechanisms and targeted therapy opportunities. To evaluate the value of anti PI3K strategy in GI cancer, different lines of GI cancer organoids were established. The organoids recapitulated the morphologies of the original tumors. Organoids were generally insensitive to PI3K inhibitors. However, CRC-3 and GC-4 showed response to mTOR inhibitor Everolimus, and GC-3 was sensitive to PI3Kδ inhibitor Idelalisib. The CRC organoid with a PIK3CA mutation showed greater sensitivity to the PI3K inhibitor Alpelisib than wildtype organoids, suggesting potential treatment options for the corresponding patients.

**Conclusion:**

Liquid biopsy holds significant promise for improving precision treatment and tumor prognosis in colorectal cancer patients. The combination of biomarker-based drug prediction with organoid-based functional drug sensitivity assay may lead to more effective cancer treatment.

**Supplementary Information:**

The online version contains supplementary material available at 10.1186/s12967-023-04675-6.

## Introduction

Liquid biopsy is a diagnostic technique that aims at sampling the body fluids rather than the tissue-of-interest itself, to infer the characteristics of the remote tissue. At present, liquid biopsy mainly includes circulating tumor cells, circulating tumor DNA, exosomes, microRNAs, circulating RNA, tumor platelets and tumor endothelial cells. Extensive research has demonstrated that tumor-derived DNA can be detected in the circulation of cancer patients in the form of circulating cell-free DNA (cfDNA). The cfDNA released from tumor cells is called circulating tumor DNA (ctDNA). ctDNA constitutes a considerable proportion of cfDNA, which could range from 0 to more than 90% [1]. Liquid biopsy of cfDNA is a good alternative to traditional biopsy procedures when the tumor tissue is difficult to sample, or when there are multiple heterogeneous tumors. The same to the traditional biopsy, liquid biopsy can reflect the genomic content of the tumor, but much more convenient and safer. In addition, liquid biopsy can also reflect the spatial and temporal heterogeneity of tumors and give a more accurate overview of mutations in the tumor [2]. Because of those progresses in liquid biopsy, the US FDA has approved several commercial kits for cancer diagnosis [3].

A major application of cfDNA liquid biopsy is to find treatment targets or therapeutic biomarkers, which can be used to guide the selection of therapeutic drugs [4]. This is especially useful for late-stage cancer patients, who have been extensively treated and exhausted all standard treatments. Liquid biopsy of cfDNA can unveil the full spectrum of genetic variants in the primary as well as metastatic tumors from the patients, whereas avoiding repeated invasive tumor biopsies [5]. In TARGET study, which aims at evaluating ctDNA for cancer patient care, ctDNA data showed good concordance with matched tumor and actionable mutations were identified in 41 of 100 patients, and 11 of these patients received a matched therapy [6]. In addition, cfDNA sequencing can be used to predict tumor mutation burden (TMB) [7] and microsatellite instability (MSI) [8], which are important markers for cancer immunotherapy. Furthermore, liquid biopsy of BRAF V600E mutation can be suggestive of resistance after melanoma patient receiving Vemurafenib treatment [9]. However, in most cases, cfDNA sequencing can hardly find meaningful gene mutations that are suggestive of certain drugs. This is a common problem for the biomarker-based precision medicine. More importantly, the accuracy of the biomarker in predicting drugs is also questionable. A large part of patients are insensitive to selected drugs based on biomarkers. This is a big problem for patients who have and received many different regimes of chemotherapy who have developed multi-drug resistance.

The application of liquid biopsy in tumor burden monitoring has been highlighted by recent discoveries. Research has shown that the level of ctDNA is closely related to the tumor stage or tumor burden. For example, in Ewing sarcoma and osteosarcoma, the detection of ctDNA as indicated by cancer specific translocations like EWSR1, was associated with more advanced stage of cancer and poorer overall survival [10]. Similarly, the level of ctDNA was highly correlated with the tumor size in non-small-cell lung cancer. Tumor burden estimated by liquid biopsy also predicted treatment response and long-term prognosis in breast cancer patients [11]. In addition, ctDNA testing also allowed for earlier response assessment than radiographic approaches [12]. Recently, low pass whole-genome sequencing (WGS) of cfDNA, which requires significantly less depth of sequencing, has been shown to be very effective at monitoring the status of breast cancer [13] and prostate cancer [14]. This is more cost-effective than classic liquid biopsy based on deep sequencing.

Liquid biopsy also holds great promise for cancer diagnosis, including early diagnosis of pre-clinical cancer and monitoring for cancer recurrence. Early diagnosis of cancer is more efficient in reducing cancer mortality than developing new drugs [15]. Tumorigenesis is a multi-stage process, where mutations are generated and accumulated even in the earliest stage. Because of its high sensitivity, liquid biopsy can be used to detect low levels of ctDNA and diagnose cancer at an early or super-early stage. In one study, liquid biopsy of cfDNA together with several protein markers, could diagnose cancer originating from several organs. The test achieved sensitivities ranging from 69 to 98% and a specificity greater than 99% [16]. At the same time, many companies are trying to develop and commercialize liquid biopsy technologies for early tumor diagnosis [17]. Liquid biopsy also offers the opportunity for tumor monitoring. For example, ctDNA analysis after surgery is a prognostic marker in stage III colon cancer and post chemotherapy ctDNA analysis may define a patient subset that remains at high risk of recurrence despite completing standard adjuvant treatment [18]. Overall, cfDNA is under intense research and more discoveries are rolling out every month, which may change the management of cancer in the future.

Liquid biopsy has been studied extensively in colorectal cancer. It had been shown to be able to detect the cause of resistance in anti-EGFR treatment. One study found that about 5–6 months following anti-EGFR antibody treatment, mutant KRAS can be detected in cfDNA [19]. This result was confirmed by another study, which revealed almost all mCRC patient developed mutations in mitogen-activated protein kinase pathway, where KRAS plays a key role [20]. In another study, late-stage CRC patients were repeatedly monitored by cfDNA testing and CT scan. Personalized ddPCR assays were designed to detect somatic structural variants (SSV) in each patient. The results showed that liquid biopsy can predict tumor recurrence or progression with 100% sensitivity and specificity. More strikingly, liquid biopsy provided 2–15 months’ lead time on detection of metastatic recurrence compared to conventional follow-up [21].

In the current study, we tried to investigate how liquid biopsy of cfDNA could benefit general cancer patients. A consecutive cohort of patients with colorectal cancer or other GI tumors were enrolled. Liquid biopsy of pre-treatment cfDNA revealed common mutations found in other sequencing project. Notably, we revealed exceptionally high allele frequency of ctDNA mutations in several patients with metastatic colorectal cancer. In addition, GI cancer organoids were established and anti-PI3K drugs were evaluated in CRC.

## Methods

### Patient

Patients with an admission diagnosis of colorectal cancer or gastrointestinal tumors were evaluated. Patients with no prior treatment for their cancer were candidates of this study. From July to October of 2017, sixty-seven patients were enrolled in the study. All participating patients gave their informed consent. The diagnosis of each patient was confirmed through endoscopic biopsy or tumor resection and subsequent histologic analysis. Metastasis were diagnosed by biopsy of tumor lesion, CT, and/or ultrasound scanning.

### Sample

One 7.5 ml blood sample was collected using EDTA tubes and processed immediately. The plasma was isolated by a two-step centrifugation method. The whole blood was first centrifuged at 1600 g for 10 min and the plasma was transferred into 2 ml EP tubes. Then the plasma was centrifuged at 16,000 g for 10 min and the supernatant was transferred into a new EP tube. The plasma samples were then stored at – 80 °C for later processing. DNA was purified from 4 ml plasma using a MagMAX Cell-Free DNA Isolation Kit (Cat. A29319), according to the manufacturer’s instructions. DNA was suspended in 40 μl water. DNA quantity and quality were assessed using the Qubit™ photometer (Life Technologies) according to the manufacturer’s instructions.

### Design of targeted sequencing

The Oncomine™ Colon cfDNA Assay kit (Cat. A31182, Thermo Fisher Scientific, US) was used for library preparation in this study [22]. This assay applies the AmpliSeq^TM^ technology to specifically capture the targeted regions of interest [23]. Single nucleotide variants and short indels that are frequently mutated in colorectal cancer are covered in this assay. Totally 48 amplicons mapping to fourteen genes are generated by this assay. These genes are: AKT1, BRAF, CTNNB1, EGFR, ERBB2, FBXW7, GNAS, KRAS, MAP2K1, NRAS, PIK3CA, SMAD4, TP53, APC. More than 240 hotspots of these genes are covered. Nine oncogenes, including EGFR, ERBB2, KRAS, BRAF, NRAS, MAP2K1, PIK3CA, AKT1, and CTNNB1, whose mutations are essential to targeted therapy, were included in the panel. Five tumor suppressor genes, including TP53, APC, FBXW7, SMAD4, and GNAS were also on the list. The details of this assay and the amplicons covered can be found on the manufacturer’s website (https://www.thermofisher.com/order/catalog/product/A31182).

### Liquid biopsy and next generation sequencing

The sequencing step was outsourced to Guangzhou Darui Biotechnology Co., Ltd. (China). 20 ng cfDNA was used as input for library preparation following the manufacturer’s protocol. Libraries were barcoded with the Ion Xpress Barcode Adapters 1–16 Kit (Thermo Fisher Scientific, US). Barcoded libraries were combined to a final concentration of 100 pM. Template preparation by emulsion PCR (emPCR) was performed on the Ion OneTouch 2 system (Thermo Fisher Scientific, US). Next-generation sequencing (NGS) was performed on the Ion PGM™ System. The depth of NGS was projected to be 20,000 ×, which enables the detection of rare variants of as low as 0.05% in allele frequency.

### Sequencing data analysis

The sequencing data was analyzed with the Torrent Suite™ Software v5.2.1. Mutations were called using the variantCaller plugin. The Ion Reporter suite (Thermo Fisher Scientific, US) was used to filter variants. A vcf file containing variants of all samples was exported for downstream analysis. The variants were then annotated using the Oncotator API (http://portals.broadinstitute.org/oncotator/) [17].

### Statistical analysis

Data analysis was carried out using R statistic software (R 3.5). Mutation data was analyzed using maftools package. When making lollipop plot, the lollipopPlot function from the maftools package was applied. Continuous data was described by mean ± SD, whereas qualitative data was described by frequency. T-test was used to compare means between two groups, whereas ANOVA test was used to for more than two groups. p values were two-sided, and p ≤ 0.05 was considered statistically significant.

### Pathology analysis

Organoid and tumor tissue were fixed in 10% neutral buffered formalin for 24 h. Samples were subjected to gradual dehydration in increasing concentrations of ethanol (75%, 85%, 95% and 100%) and embedded in paraffin blocks. 2 mm sections were made for paraffin blocks. Slices were stained using hematoxylin and eosin (H&E). The stained sections were dehydrated by pure alcohol and made transparent by xylene. Slices were covered with mounting medium and sealed with coverslips.

### Organoid culture

Tumor specimens are removed and stored in DMEM medium and transported to the laboratory at 4 °C. Tissue was fragmented and washed in PBS with 50 U/ml penicillin–streptomycin (Thermo Fisher). Then tissue was digested for 2 h in 1 ml digestion solution containing 1 mg/ml collagenase IV (Sigma-Aldrich), 0.1 mg/mL DNase I, and 10 μM Y-27632. After completion of digestion, tissue fragments were sedimented and the supernatant was collected. Cells were spun down and washed with DMEM medium for three times. Finally, cells were mixed with Matrigel (Corning^®^ Matrigel) and seeded into a 96-well plate. Colorectal cancer organoid medium was added in each well after Matrigel solidifying. The composition of colorectal cancer organoid medium had been published by the laboratory of Hans Clevers. The basal medium contained Advanced DMEM/F12, Glutamax, HEPES, penicillin–streptomycin, B27 (Life Technologies), N2 (Invitrogen), N-acetylcysteine (1 mM; Sigma), Nicotinamide (10 mM; Sigma). The basal medium was supplemented with growth factors, including human EGF (50 ng/ml), Noggin (100 ng/ml) and R-spondin (500 ng/ml), and small molecules, including CHIR99021 (3 μM; Millipore) and valproic acid (1 mM; Sigma), A83-01 (500 nM; Tocris), SB202190 (10 μM; Sigma), Prostaglandin E2 (10 nM; Tocris); Gastrin (10 nM; Tocris), and Y-27632 (10 μM; Abmole). Organoids were passaged about 2 times each week.

### Drug screening with organoid

Organoids were removed from cell culture plate and digested in TrypLE express enzyme (Gibco™, USA) in a shaker (Topscien, CN) at 37 °C for 10 min. Then cells were spun down and resuspended in organoid culture medium containing 5% Matrigel. Next, cells were dispensed into 384-well plates (GREINER BIO-ONE, 781091) at 1000 cells/25 μl medium/well using an automated reagent dispenser (Biotek multiflo). After 24 h of plating, Alpelisib (TargetMol) and Everolimus (TargetMol) were dispensed at six concentrations (10 μM, 3 μM, 1 μM, 0.4 μM, 0.12 μM, and 0.04 μM) in 3 replicates using Acoustic Liquid Handlers (Labcyte echo 650). 0.1% DMSO (Sigma-Aldrich) and 4 μM MG132 (MCE) were used as negative and positive controls, respectively. After 72 h, 10 μl CellTiter-Glo^®^ Luminescent Cell Viability Assay reagent was dispensed into each well of the 384-well plate and the luminance signals were read using a MicroplateReader (Thorme Fisher). Drug screening data were analyzed using Prism GrapdPad 9.0.

### RNA sequencing

CRC-1 organoid was treated with either 4 μM Alpelisib or DMSO for 48 h. Three replicates were employed for each group. Then cells were harvested with TRIzol™ Reagent (Thermo Fisher Scientific). Whole transcriptome sequencing was outsourced to GUANGZHOU IGE BIOTECHNOLOGY LTD (China). Raw reads were quality checked using FastQC. Reads were aligned to the reference genome using Hisat2. Differential expression analysis was performed using the limma package. GSEA analysis was performed using the ClusterProfiler package in R.

## Results

### Clinical and pathological characteristics of the patients

To evaluate how liquid biopsy could benefit gastrointestinal cancer patients, we prospectively enrolled 67 consecutive treatment-naive patients. The characteristics of patients were summarized in Table 1. There were 58 colorectal cancer (CRC) and 3 gastric cancer patients. The other 6 patients had benign colon polyps (4), colitis (1) or stomach GIST (Gastrointestinal Stromal Tumor) (1). In particular, 17 patients with distant metastasis (stage IV) were included in the study. There were more male patients than female patients (47 vs 20), and the average age of the patients was 61.7 ± 10.9 years. There were similar numbers of colon and rectum cancer patients (28 vs 30). Most of the colorectal cancer were moderately differentiated (Table 1). One blood sample from each patient was tested by cfDNA (circulating free DNA) sequencing.

**Table 1:** Clinical and pathological characteristics of the patients

	All (67)	CRC (58)
Gender		
Male	47	41
Female	20	17
Age	61.7 ± 10.9	62.8 ± 9.8
Location		
Colon	31	28
Rectum	32	30
Stomach	4	
Pathology		
Adenocarcinoma	61	58
GIST	1	
Non-malignant	5	
Differentiation		
Moderately	33	32
Poorly	13	11
Mucinous	4	4
Stage		
0	1	1
I	7	7
II	16	16
III	20	19
IV	17	15
Other	6	

*CRC* colorectal cancer

### Overview of the results of cfDNA liquid biopsy analysis

**Fig. 1:**
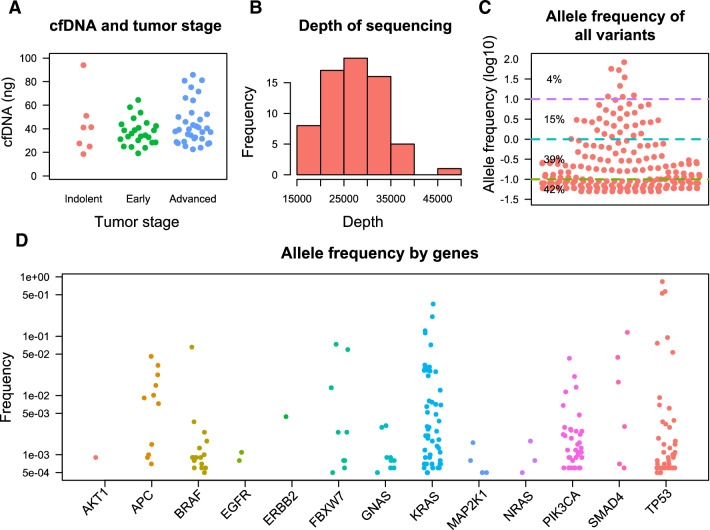
Overview of the results of cfDNA liquid biopsy. **A** the quantity of extracted cfDNA grouped by tumor types. **B** Histogram showing the distribution of the depth of sequencing across all samples. **C** Allele frequency of mutant across all samples. The percentage in the plot represent the proportion of variants within each range of allele frequency. **D** Allele frequency of each mutant grouped by gene. CTNNB1 was wildtype in all tested samples and was not shown in the plot

The average yield of extracted cfDNA from 10 ml venous blood was 41.6 ± 16.8 ng. No significant difference was observed in yield of cfDNA in patients with benign, early stage and late-stage diseases (Fig. 1A). The average depth of sequencing was 26,997 ± 5937, which was larger than preplanning (20,000 ×) (Fig. 1B). Using a pre-defined cutoff value of allele frequency (0.05%) for variant filtering, 195 mutations were found in 58 samples, whereas no mutation was identified in the rest 9 samples. The allele frequency of mutants in different samples varied, with 81% of mutants less than 1% and 42% of mutants less than 0.1% (Fig. 1C). Similarly, the allele frequency of mutants also varied across different genes (Fig. 1D).

### Mutations detected by liquid biopsy

**Fig. 2:**
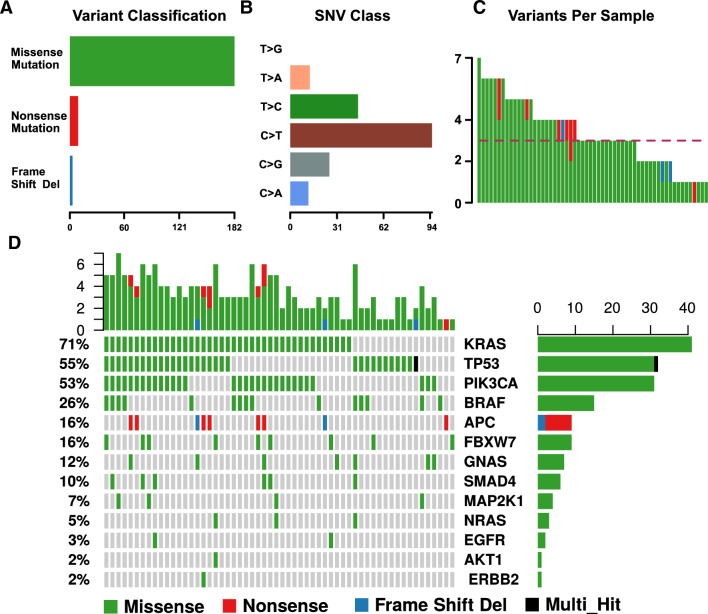
the features of variants detected by cfDNA liquid biopsy. **A** barplot showing the proportion of each type of mutation detected by liquid biopsy. **B** Mutation signature of all variants detected in liquid biopsy. **C** Barplot showing the number of mutations detected in each sample. **D** Oncoprint plot showing all variants detected in each gene and sample

Most of the variants were missense mutations. Nonsense mutations were detected exclusively in APC. In addition, frame shift deletions were discovered in APC and TP53. Most variants were transition mutations from C to T or T to C (Fig. 2B). Transversion mutation from C to G was also common (Fig. 2B). The median number of variants detected in each sample was 3 (range: 1–7) (Fig. 2C). Thirteen genes had at least one mutation. KRAS mutation was most popular, occurring in 71% of samples with positive mutations (41 out of 58). This was followed by TP53 and PIK3CA, both of which occurred in more than 50% of samples. (Fig. 2D).

**Fig. 3:**
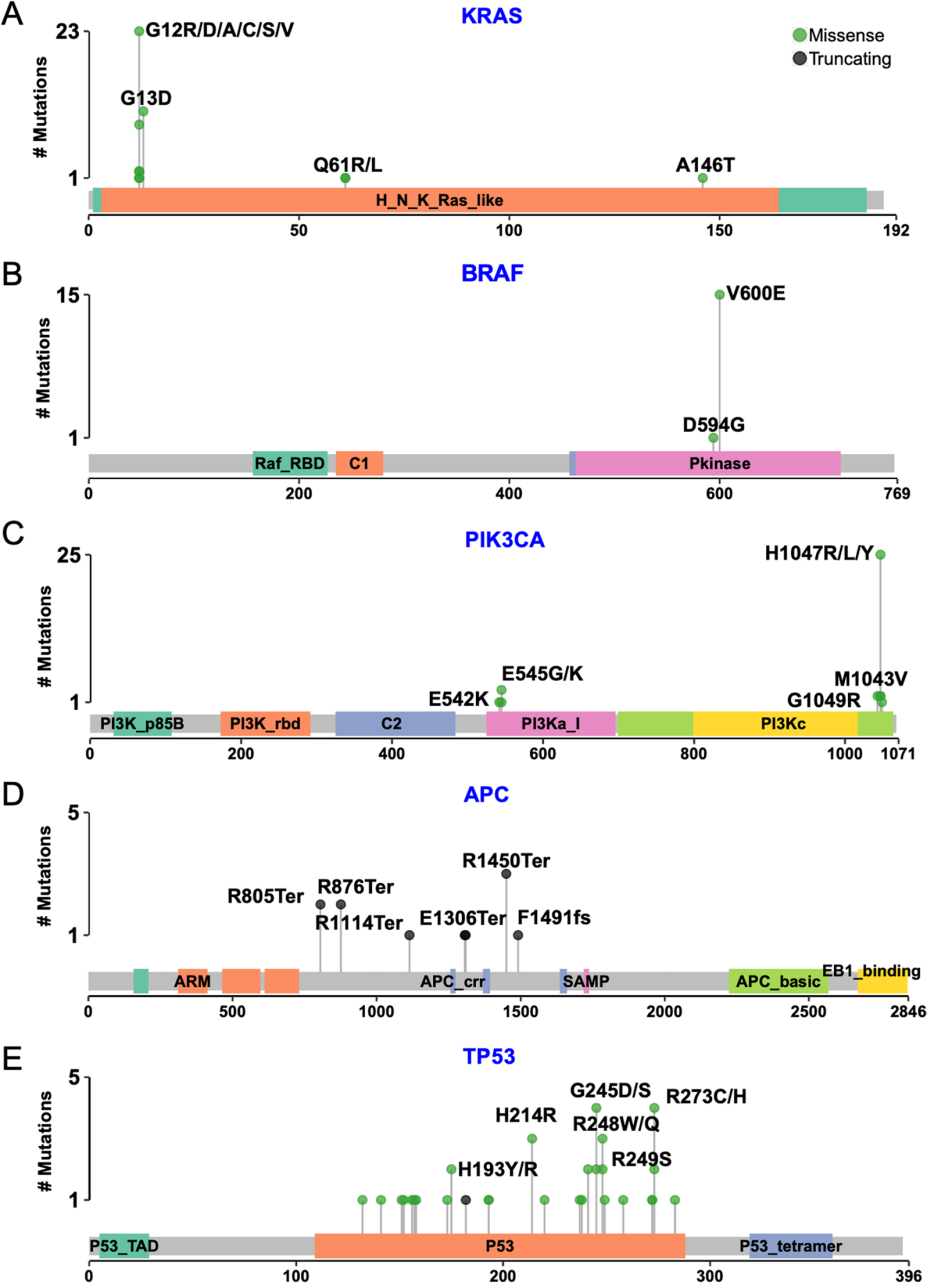
lollipop plots showing the location of mutations in each gene. The grey bar represents the whole length of the protein, and the colored boxes are functional domains of each protein. The height of vertical lines represents a total number of mutations identified in each amino acid. **A** to **E**, mutations detected in KRAS, BRAF, PIK3CA, APC and TP53, respectively

KRAS mutations occurred mainly in two hotspots, i.e., codon 12 and 13. Rarer KRAS hotspot mutations were found in codon 61 and 146 (Fig. 3A) [24]. BRAF acts downstream of RAS and is frequently mutated in cancers like melanoma. In this cohort, 16 BRAF variants were found in 15 samples, of which 6 samples (10.3% of mutation-positive samples) had allele frequencies larger than 0.1% (Fig. 3B). All BRAF variants were p.V600E with only one exception that was p.D594G. The PIK3CA gene encodes p110α protein, which is a subunit of PI3K. There were two amplicons targeting two hotspots in PIK3CA (E545 and H1047). Totally 31 PIK3CA mutations were identified, most of which were H1047 (Fig. 3C).

TP53 and APC were top two highly mutated tumor suppressor genes identified in this study. Different from the oncogenes listed above, no obvious mutation hotspots were found in TP53 or APC (Fig. 3D and E). All variants detected in APC were truncating mutations, including nonsense mutations and frame shift deletions. In contrast, almost all TP53 variants detected were missense mutations. These data agreed with previous studies [25].

### High allele frequency detected in liquid biopsy associated with later tumor stage

**Fig. 4:**
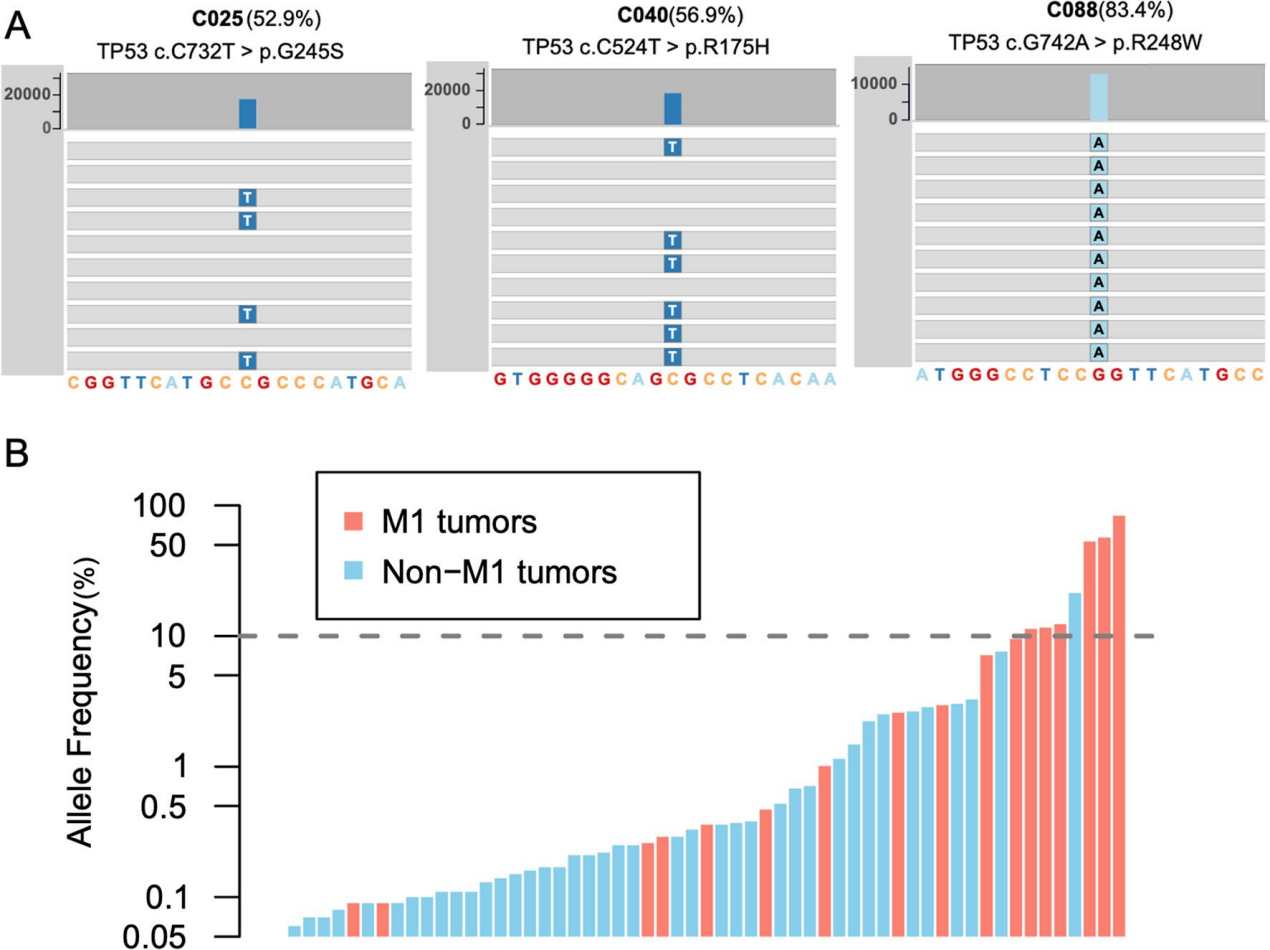
Mutations with high allele frequency discovered in liquid biopsy. **A** Visualization of three TP53 mutations with high allele frequency using a genome browser. **B** Barplot showing the association between the highest allele frequency of mutations detected in each patient with their tumor metastasis status. Red bars represent patients with distant metastases, whereas blue bars represent patients without distant metastases. The y axis was in log10 scale

Although most of the mutations had an allele frequency less than 1%, 8 variants from 7 samples showed high allele frequencies which were larger than 10% (Table 2). 2 of those 8 variants came from the same patient (Case C25). The genes with high allele frequencies were KRAS, TP53 and SMAD4, with 4, 3 and 1 mutations detected, respectively. Noteworthy, 3 samples showed allele frequencies larger than 50% (Table 2). All these mutations came from TP53, which were hot spots mutations affecting R175, G245, and R248 amino acid residues of the DNA binding motif of p53 (Fig. 4A). Upon scrutinizing, we found that 6 out of the 7 patients with exceptionally high allele frequency mutations had metastatic diseases, including 3 liver metastasis, 2 lung metastasis and 1 peritoneal metastasis (Table 2). However, Case C065 was an exception with a 2.5 cm polys-like rectal adenocarcinoma with submucosa invasion (pT1NxM0, stage I). However, the KRAS G12S mutation detected in this patient had a high allele frequency (21.37%), disproportional to the small tumor size and early tumor stage (Table 2). Of note, this study enrolled 6 patients with non-cancerous lesions, including 4 polyps. 3 out of 4 patients were confirmed to carry colorectal adenomatous polyps. Mutations were found in all three patients (Additional file 1: Table S1). The fourth patient was a young male with P-J syndrome (Additional file 1: Fig. 1), which is caused by germline mutation of STK11 (data not shown). These data suggest ctDNA is also actively released from early phase tumors. Dozens of hamartoma polyps were discovered throughout the colon and rectum of this patient (Additional file 1: Figure S1A, 1C and 1E). In addition, a large polypoid polyp of 4 cm in diameter was found in the sigmoid colon of this patient (Additional file 1: Figure S1B, 1D and 1F) (Table 3).

**Table 2:** Mutations with exceptional high allele frequency

ID	Gender/Age	Location	Stage	Metastasis	DNA	Protein	dbSNP	AF (%)
C004	M/73	Sigmoid	IV (cT4N2M1)	Liver	chr18:48591904 C > T	SMAD4 p.P356L		11.61
C025	M/69	Sigmoid	IV (pT4N2bM1)	Peritoneal cavity	chr17:7577548 C > T	TP53 p.G245S	rs28934575	52.92
C025	M/69	Sigmoid	IV (pT4N2bM1)	Peritoneal cavity	chr12:25398284 C > G	KRAS p.G12A	rs121913529	34.94
C031	F/55	Rectum	IV (pT4aN0M1)	Lung	chr12:25398284 C > G	KRAS p.G12R	rs121913529	12.38
C040	M/68	Sigmoid-Rectum	IV (cT4aNxM1)	Lung	chr17:7578406 C > T	TP53 p.R175H	rs28934578	56.86
C048	M/52	Sigmoid	IV (cTxNxM1)	Liver	chr12:25398284 C > G	KRAS p.G12R	rs121913529	11.37
C065	F/75	Rectum	I (pT1NxM0)		chr12:25398284 C > T	KRAS p.G12S	rs121913529	21.37
C088	M/60	Sigmoid-Rectum	IV (cTxNxM1)	Liver	chr17:7577539 G > A	TP53 p.R248W	rs121912651	83.42

**Table 3:** Mutations detected in a patient with P-J syndrome

Locus	Genotype	Ref	Genes	Protein	AF (%)
chr3:178952085	A/G	A	PIK3CA	p.H1047R	0.11
chr12:25398281	C/T	C	KRAS	p.G13D	0.81
chr12:25398284	CC/CG	CC	KRAS	p.G12R	2.86
chr17:7578208	T/C	T	TP53	p.H214R	0.69

### Establishment and characterization of GI cancer organoids

**Fig. 5:**
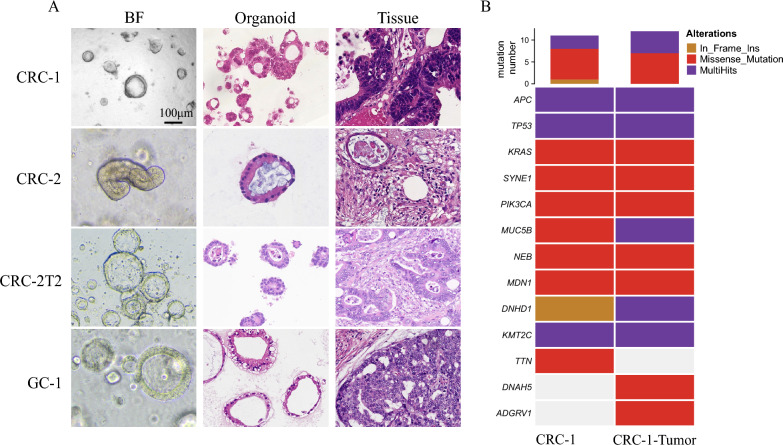
Establishment and characterization of colorectal cancer organoids. **A** Light microscopic (left column) and HE staining of four lines of organoids (middle column) and the corresponding tumor tissue (right column). HE, Hematoxylin–eosin staining; BF, Bright field. Scale bar 100 μm. **B** Non-synonymous mutated genes detected in CRC-1 and paired tumor tissue (CRC-1-Tumor). MultiHits indicates multiple mutations occur in the gene

PIK3CA mutation was found in multiple cfDNA samples in this study. PI3K pathway inhibitors have been approved for treating breast cancer by FDA [26]. To explore the potential of targeting PI3K in CRC, we took advantage of patient-derived organoid models, which can faithfully recapitulate the genetic background and phenotypes of cancer. Several lines of gastrointestinal (GI) cancer organoids were successfully established using tumor samples. The morphology of the organoids was similar to the corresponding tumor tissue (Fig. 5A). To identify therapeutic targets and check whether the organoids carry identical gene mutations as the primary tumor, we conducted whole exome sequencing (WES). 659 non-synonymous mutations were found in CRC-1-Tumor and 650 in CRC-1 organoid. Mutations in PIK3CA, APC, TP53, KRAS, SYNE1, NEB, and MDN1 were observed in both the original cancer tissue and the organoid (Fig. 5B). A PIK3CA p.E542K mutation was identified in CRC-1 with an allele frequency of 57.5%, suggesting possible founding mutation during early tumor formation stages (Table 4). No PIK3CA mutations were identified in CRC-2, CRC-2T2 or GC-1 (Table 4).

**Table 4:** PIK3CA status of organoid lines detected by Next Generation Sequencing

Organoid	PIK3CA status	DNA	cDNA change	AA change	Allele frequency
CRC-1	Mutated	chr3 179,218,294 G to A	c.G1624A	p.E542K	57.5%
CRC-2	Wildtype	–	–	–	–
CRC-2T2	Wildtype	–	–	–	–

### Evaluating anti-PI3K strategy in GI cancer organoids

**Fig. 6:**
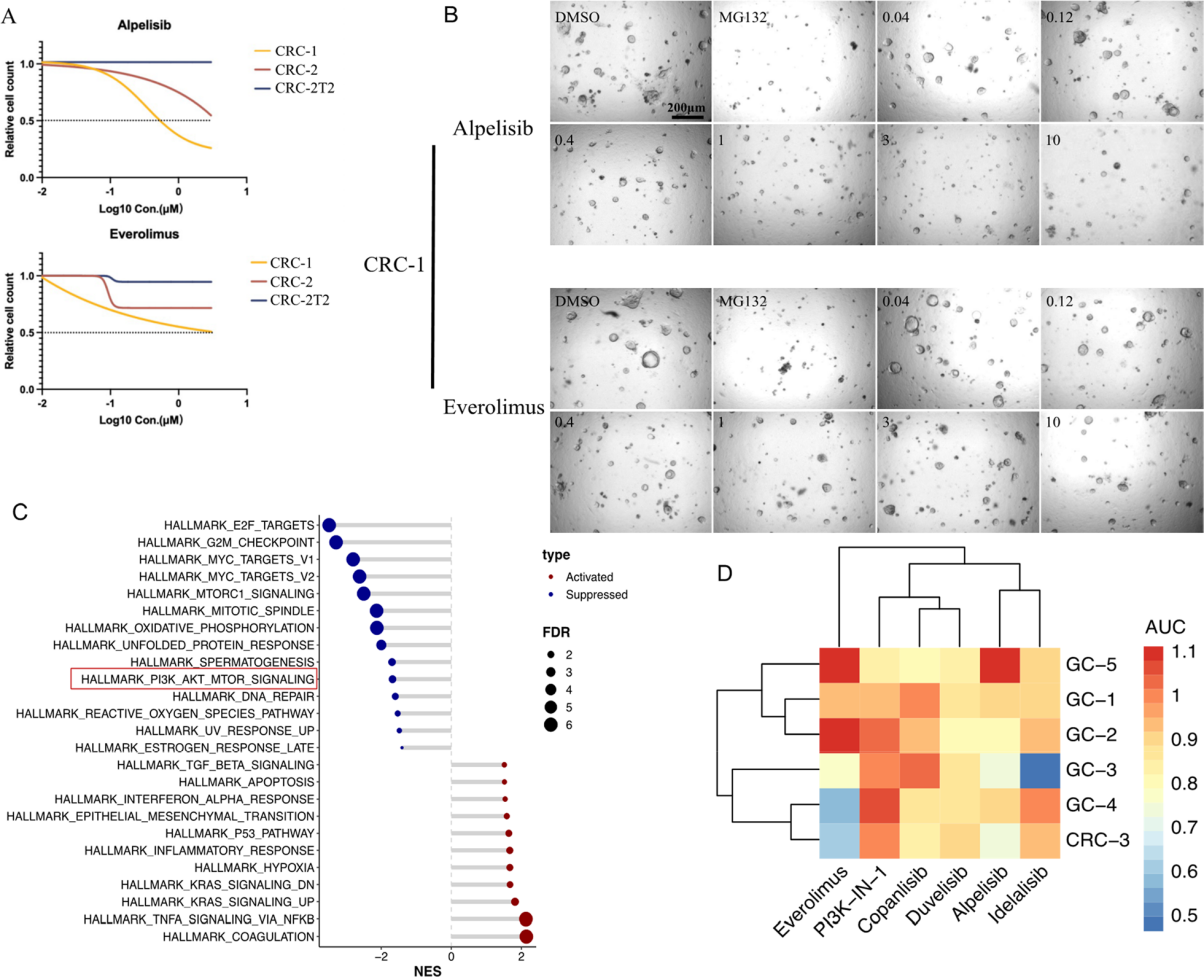
Screening of PI3K and mTOR inhibitors in organoids. **A** Dose–response curve showing the response of organoids to Alpelisib and Everolimus treatment. **B** Light microscopic photos showing Alpelisib and Everolimus treatment of CRC-1. Scale bar 200 μm. **C** Lollipop plot showing the top HALLMARK signaling pathways associated with Alpelisib treatment according to GSEA analysis. The horizontal coordinate is the Normalized Enrichment Score (NES) and the vertical axis shows the significant gene sets, with red dots for activation and blue for inhibition. FDR, false discovery rate. **D** Heatmap showing the effect of PI3K inhibitors on a panel of 6 lines organoids with wildtype PI3K. AUC, area under the curve of the dose–response curve

To evaluate the potential of targeting PI3K pathway in GI tumors, patients-derived cancer organoids were applied. We firstly tested PI3Kα inhibitor Alpelisib and mTOR inhibitor Everolimus, which are two PI3K pathway inhibitors approved for cancer treatment [26]. Alpelisib inhibited the viability of CRC-1, which carried PIK3CA p.E542K mutation, in a dose-dependent manner (IC50 = 0.34 μM) (Fig. 6A and B). However, the control organoids CRC-2 and CRC-2T2, which had wildtype PIK3CA showed resistance to Alpelisib (Fig. 6A). Similarly, CRC-1 also appeared to be more sensitive to mTOR inhibitor Everolimus than CRC-2 and CRC-2T2 organoids (Fig. 6A). To validate the effect of Alpelisib on tumor cells, whole transcriptomic analysis was performed comparing Alpelisib treated versus control organoid (Additional file 1: Figure S2). GSEA analysis of Hallmark gene sets revealed that Alpelisib treatment of CRC-1 led to down-regulation of cell circle and cell proliferation-related signals, like E2F targets, G2M checkpoints, MYC targets, etc. (Fig. 6C and Additional file 1: Figure S2). At the same time, PI3K/AKT/MTOR signaling was also down-regulated, confirming the effects of Alpelisib on tumor cells (Fig. 6C). Alpelisib has been recommended for breast cancer patients carrying PIK3CA mutations. Our results revealed that Alpelisib was also effective in PIK3CA mutated CRC organoids, suggesting the consistency between biomarker-based and organoid-based precision treatment.

As many other inhibitors targeting different PI3K isoforms have been developed for lymphohematopoietic malignancies or non-malignant diseases, we expanded our test to include these inhibitors to explore potential off-label use. Six PI3K signaling inhibitors, including Alpelisib (PI3Kα), Copanlisib (PI3Kα and PI3Kδ), Duvelisib (PI3Kδ and PI3Kγ), Idelalisib (PI3Kδ), Everolimus (mTOR) and PI3K-IN-1 (mTOR and PI3Kγ) were selected. Six GI organoids with wildtype PI3K were chosen for this panel screening. Organoids were generally insensitive to PI3K/mTOR inhibitors (Fig. 6D). However, CRC-3 and GC-4 showed response to Everolimus but not Alpelisib (Fig. 6D). Similarly, GC-3 was sensitive to PI3Kδ inhibitor Idelalisib. These data suggested some GI tumors with wildtype PI3K may be candidates for anti-PI3K/mTOR treatment and organoid-based functional drug screening may reveal sensitive drugs for biomarker-negative tumors.

## Discussion

Liquid biopsy of cancer is gaining an increasing popularity in recent years. In this study, we applied ultra-deep targeted next generation sequencing (NGS) to a cohort of consecutively enrolled GI cancer patients. The 14 genes included in this panel are either targetable themselves or may affect the response of tumor to certain drugs. The results of liquid biopsy including mutation rate and allele frequency generally agreed with previous studies. It gave us a deeper understanding of the value of liquid biopsy in the management of patients with GI cancers.

An important application of liquid biopsy is for tumor diagnosis, including evaluating tumor burden, identifying residual, recurrent and early stage tumors [2]. For example, a study in breast cancer showed that ctDNA levels correlated with tumor burden and worked better than CA15-3 or circulating tumor cells [27]. In this study, we observed exception high allele frequency variants in certain patients. Upon scrutiny, we found metastatic tumors in most of those patients. This linkage may help to explain the occasionally encountered high allele frequency variants in liquid biopsy. These high allele frequency variants may predict unfavorable outcomes of the patients. However, because of the short follow-up time in this study, we didn’t observe enough number of incidences like tumor recurrence or death of patients related to high allele frequency. A longer observation may see the separation of survival curves between those two groups with or without high allele frequency variants in the future.

The most clinically relevant utility of liquid biopsy is to guide targeted therapy in cancer patients. EGFR, RAS family and BRAF are closely related to anti-EGFR therapy [19, 28, 29, 30]. In this study, KRAS was the most frequently mutated gene detected. BRAF mutation was identified in 22.4% (15 out of 67) samples in this cohort, which is higher than CRC tissues (9%) [25]. BRAF can be targeted by inhibitors such as Vemurafenib, which is recommended in patients with BRAF mutated tumors who were not previously treated with an EGFR inhibitor [31, 32]. High rate of PIK3CA mutation was also discovered in CRC patients in this study, occurring in 46.3% (31 out of 67) samples [25]. PI3K inhibitor has been approved in breast cancer treatment [26]. The mutations detected in this study are suggestive of possible tumor progression mechanisms and opportunities of targeted therapy.

Classic precision medicine mainly relies on genetic testing like next generation sequencing for biomarker discovery and treatment assignment. However, this strategy is limited by the fact that druggable targets can only be identified in a small percentage of patients. In addition, biomarker-guided precision medicine is also restricted by low sensitivity rate after patients receiving the assigned treatment. Organoids is a high-fidelity model of human cancer and can be used to test whether patients can benefit from certain drug treatment. The consistency between organoid-based drug tests and the real human response has been validated by various studies. Thus, the combination of biomarker-based drug prediction with functional drug sensitivity assay using patient-derived organoid may increase the chance of more effective cancer treatments. In this study, we test PI3K pathway inhibitors in organoids with different PI3K mutation status identified by sequencing. We found that the CRC organoids carrying PIK3CA mutation was more sensitive to PI3K inhibitor and mTOR inhibitor than the organoids with wildtype PIK3CA. This result demonstrates the consistency between sequencing and organoid directed precision cancer treatment.

In addition, we also found that a small number of organoid lines responded to certain PI3K signaling inhibitors without guidance of biomarkers. One GI cancer organoid is quite sensitive to PI3Kδ inhibitor Idelalisib, but not to other pan PI3K inhibitors, including Copanlisib and Duvelisib. Idelalisib has been approved by FDA for treating several subtypes of lymphoma [33]. Similarly, the mTOR inhibitor Everolimus is also effective in two organoid lines. The underlying molecular mechanisms are unclear yet. More mechanistic studies are required to address this question in the future. These data suggest that organoid-based functional drug screening may reveal sensitive drugs for biomarker-negative tumors. Drug re-purposing and off-label use are important directions to expand the arsenal of anti-cancer drugs. Traditional clinical trials are inefficient in these situations given the scope of candidate drugs and limited availability of suitable patients. Organoid could be a good tool for such applications. However, we also noted that more clinical data are needed to validate the value of organoid-based precision medicine.

## Conclusion

This study confirmed the value of liquid biopsy in the prognosis and treatment of CRC. It also demonstrated the potential of targeting PI3K signaling in GI tumors. This study serves as proof-of-concept for combining liquid biopsy and organoid-based in-vitro drug screening for precision cancer treatment.

### Supplementary Information

**Additional file 1. Figure S1.** eEarly-stage tumors identified in a patient with P-J syndrome.. Colonoscopy diagnosis and HE staining of the tumors from a PJS patient. A and B, colonoscopy images showing the polyps (A) and the bulk tumor (B) detected in the ascending colon and sigmoid colon, respectively. C and D, HE staining of the biopsies from A and B at 40x magnification, respectively. E and F, HE staining of the biopsies from A and B at 100x magnification, respectively. The white circles in the plots indicate polys or tumors found under colonoscopy. The scale bars in the plots stand for 625 μm (C and D) or 200 μm (E and F). **Figure S2.** RNA sequencing analysis of organoid treated with Alpelisib. A, Volcano plot showing the differentially expressed genes comparing Alpelisib treated and control CRC-1 organoid. Genes upregulated in the treated group are depicted in orange, while those downregulated are shown in blue. B, Heatmap of top differentially expressed genes comparing Alpelisib treated and control CRC-1 organoid. C and D, GO analysis of up-regulated genes and down-regulated genes. E, F and G, Gene enrichment plots showing the PI3K_AKT_MTOR, APOPTOSIS, E2F_TARGETS pathways. **Table S1.** All mutations detected in the liquid biopsy of patients.

## Data Availability

The datasets during and/or analyzed during the current study available from the corresponding author on reasonable request.
