# Supplementary material for: Integrating cfDNA liquid biopsy and organoid-based drug screening reveals PI3K signaling as a promising therapeutic target in colorectal cancer
Source: J Transl Med. 2024 Feb 3;22:132. doi: 10.1186/s12967-023-04675-6 (PMC10837874; doi:10.1186/s12967-023-04675-6)
Supplement: Supplementary file 1 — Additional file 1. Figure S1. eEarly-stage tumors identified in a patient with P-J syndrome.. Colonoscopy diagnosis and HE staining of the tumors from a PJS patient. A and B, colonoscopy images showing the polyps (A) and the bulk tumor (B) detected in the ascending colon and sigmoid colon, respectively. C and D, HE staining of the biopsies from A and B at 40x magnification, respectively. E and F, HE staining of the biopsies from A and B at 100x magnification, respectively. The white circles in the plots indicate polys or tumors found under colonoscopy. The scale bars in the plots stand for 625 μm (C and D) or 200 μm (E and F). Figure S2. RNA sequencing analysis of organoid treated with Alpelisib. A, Volcano plot showing the differentially expressed genes comparing Alpelisib treated and control CRC-1 organoid. Genes upregulated in the treated group are depicted in orange, while those downregulated are shown in blue. B, Heatmap of top differentially expressed genes comparing Alpelisib treated and control CRC-1 organoid. C and D, GO analysis of up-regulated genes and down-regulated genes. E, F and G, Gene enrichment plots showing the PI3K_AKT_MTOR, APOPTOSIS, E2F_TARGETS pathways. Table S1. All mutations detected in the liquid biopsy of patients. [file 12967_2023_4675_MOESM1_ESM.docx]

**Additional file 1**

**Figure S1. Early-stage tumors identified in a patient with P-J syndrome.**


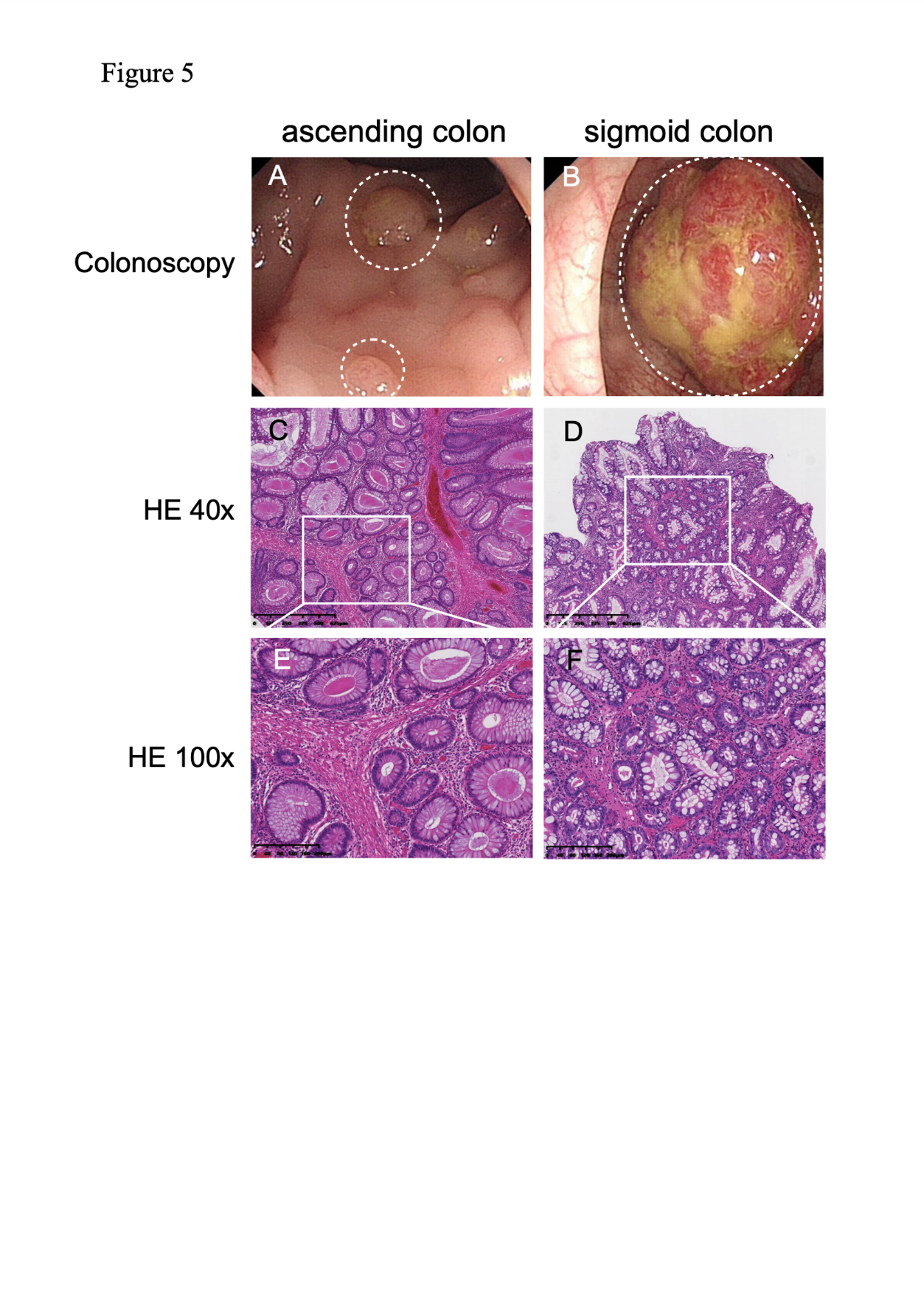


Figure S1. eEarly-stage tumors identified in a patient with P-J syndrome.. Colonoscopy diagnosis and HE staining of the tumors from a PJS patient. A and B, colonoscopy images showing the polyps (A) and the bulk tumor (B) detected in the ascending colon and sigmoid colon, respectively. C and D, HE staining of the biopsies from A and B at 40x magnification, respectively. E and F, HE staining of the biopsies from A and B at 100x magnification, respectively. The white circles in the plots indicate polys or tumors found under colonoscopy. The scale bars in the plots stand for 625 μm (C and D) or 200 μm (E and F).

**Figure S2. Pathway enrichment analysis of DEGs in Alpelisib-treated CRC organoids**

Figure S2. RNA sequencing analysis of organoid treated with Alpelisib. A, Volcano plot showing the differentially expressed genes comparing Alpelisib treated and control CRC-1 organoid. Genes upregulated in the treated group are depicted in orange, while those downregulated are shown in blue. B, Heatmap of top differentially expressed genes comparing Alpelisib treated and control CRC-1 organoid. C and D, GO analysis of up-regulated genes and down-regulated genes. E, F and G, Gene enrichment plots showing the PI3K_AKT_MTOR, APOPTOSIS, E2F_TARGETS pathways.

**Materials**

**table S1. All mutations detected in the liquid biopsy of patients.**

| **Sample.Name** | **Locus** | **Genotype** | **Ref** | **Genes** | **Protein** | **Frequency** |
| --- | --- | --- | --- | --- | --- | --- |
| C001 | chr7:140453136 | A/T | A | BRAF | p.V600E | 0.06 |
| C002 | chr17:7577568 | C/T | C | TP53 | p.C238Y | 0.06 |
| C002 | chr7:140453136 | A/T | A | BRAF | p.V600E | 0.09 |
| C003 | chr3:178952085 | A/G | A | PIK3CA | p.H1047R | 0.06 |
| C003 | chr7:140453136 | A/G | A | BRAF | p.V600E | 0.17 |
| C003 | chr12:25378562 | C/T | C | KRAS | p.A146T | 0.12 |
| C003 | chr12:25398281 | C/T | C | KRAS | p.G13D | 0.71 |
| C003 | chr12:25398284 | C/G | C | KRAS | p.G12R | 0.18 |
| C003 | chr15:66774131 | G/A | G | MAP2K1 | p.E203K | 0.05 |
| C003 | chr17:7577121 | G/A | G | TP53 | p.R273C | 0.06 |
| C004 | chr3:178952090 | G/C | G | PIK3CA | p.G1049R | 0.21 |
| C004 | chr7:140453136 | A/T | A | BRAF | p.V600E | 6.56 |
| C004 | chr12:25398284 | C/G | C | KRAS | p.G12R | 0.06 |
| C004 | chr17:7578272 | G/T | G | TP53 | p.H193Y | 0.11 |
| C004 | chr18:48591904 | C/T | C | SMAD4 | p.P356L | 11.61 |
| C005 | chr7:140453136 | A/G | A | BRAF | p.V600E | 0.06 |
| C005 | chr12:25398281 | C/T | C | KRAS | p.G13D | 0.17 |
| C005 | chr12:25398284 | C/G | C | KRAS | p.G12R | 0.07 |
| C007 | chr4:153249385 | G/A | G | FBXW7 | p.R465C | 0.05 |
| C007 | chr17:7578474 | G/A | G | TP53 | p.P151S | 0.1 |
| C007 | chr17:7578535 | T/C | T | TP53 | p.K132R | 0.06 |
| C008 | chr3:178952085 | A/G | A | PIK3CA | p.H1047R | 0.11 |
| C008 | chr12:25398281 | C/T | C | KRAS | p.G13D | 0.81 |
| C008 | chr12:25398284 | C/G | C | KRAS | p.G12R | 2.86 |
| C008 | chr17:7578208 | T/C | T | TP53 | p.H214R | 0.69 |
| C010 | chr17:7577120 | C/T | C | TP53 | p.R273H | 0.13 |
| C011 | chr3:178952085 | A/G | A | PIK3CA | p.H1047R | 0.12 |
| C011 | chr12:25398281 | C/T | C | KRAS | p.G13D | 0.77 |
| C011 | chr12:25398284 | C/G | C | KRAS | p.G12R | 2.6 |
| C011 | chr17:7578208 | T/C | T | TP53 | p.H214R | 0.61 |
| C012 | chr1:115256529 | T/C | T | NRAS | p.Q61L | 0.17 |
| C012 | chr3:178936092 | A/G | A | PIK3CA | p.E545G | 0.68 |
| C012 | chr3:178952085 | A/G | A | PIK3CA | p.H1047R | 0.12 |
| C012 | chr12:25380276 | T/A | T | KRAS | p.Q61R | 0.06 |
| C012 | chr15:66727442 | T/C | T | MAP2K1 | p.F53C | 0.16 |
| C013 | chr17:7577090 | C/T | C | TP53 | p.R283P | 0.05 |
| C014 | chr3:178952085 | A/G | A | PIK3CA | p.H1047R | 0.06 |
| C014 | chr12:25398284 | C/T | C | KRAS | p.G12D | 0.37 |
| C014 | chr17:7577570 | C/T | C | TP53 | p.M237I | 0.1 |
| C015 | chr1:115258746 | C/T | C | NRAS | p.G12D | 0.05 |
| C015 | chr4:153247289 | G/A | G | FBXW7 | p.R505C | 0.24 |
| C015 | chr12:25398284 | C/T | C | KRAS | p.G12D | 0.3 |
| C015 | chr14:105246551 | C/T | C | AKT1 | p.E17K | 0.09 |
| C015 | chr17:7578406 | C/T | C | TP53 | p.R175H | 0.33 |
| C015 | chr17:7578412 | A/G | A | TP53 | p.V173A | 0.15 |
| C016 | chr3:178952085 | A/G | A | PIK3CA | p.H1047R | 0.16 |
| C016 | chr20:57484421 | G/A | G | GNAS | p.R201H | 0.05 |
| C017 | chr12:25398284 | C/T | C | KRAS | p.G12D | 0.06 |
| C017 | chr17:7577534 | C/A | C | TP53 | p.R249S | 0.07 |
| C017 | chr17:7577547 | C/T | C | TP53 | p.G245D | 0.29 |
| C018 | chr17:7578457 | C/T | C | TP53 | p.R158H | 0.07 |
| C020 | chr7:140453136 | A/G | A | BRAF | p.V600E | 0.1 |
| C020 | chr17:7578190 | T/C | T | TP53 | p.Y220C | 0.11 |
| C021 | chr12:25398284 | C/T | C | KRAS | p.G12D | 0.52 |
| C023 | chr3:178952085 | A/T | A | PIK3CA | p.H1047L | 0.06 |
| C023 | chr7:140453136 | A/G | A | BRAF | p.V600E | 0.06 |
| C023 | chr12:25398284 | C/G | C | KRAS | p.G12R | 0.14 |
| C025 | chr7:140453136 | A/T | A | BRAF | p.V600E | 0.05 |
| C025 | chr7:140453154 | T/C | T | BRAF | p.D594G | 0.09 |
| C025 | chr12:25398284 | C/G | C | KRAS | p.G12A | 34.94 |
| C025 | chr17:7577548 | C/T | C | TP53 | p.G245S | 52.92 |
| C027 | chr3:178952085 | A/G | A | PIK3CA | p.H1047R | 0.19 |
| C027 | chr7:140453136 | A/G | A | BRAF | p.V600E | 0.09 |
| C027 | chr12:25398284 | C/G | C | KRAS | p.G12R | 0.21 |
| C028 | chr3:178952085 | A/G | A | PIK3CA | p.H1047R | 0.26 |
| C028 | chr12:25398281 | C/T | C | KRAS | p.G13D | 0.07 |
| C029 | chr3:178952085 | A/G | A | PIK3CA | p.H1047R | 0.08 |
| C029 | chr12:25398281 | C/T | C | KRAS | p.G13D | 0.07 |
| C029 | chr12:25398284 | C/T | C | KRAS | p.G12D | 0.21 |
| C029 | chr17:7578463 | C/T | C | TP53 | p.R156P | 0.06 |
| C030 | chr5:112173704 | C/T | C | APC | p.R805Ter | 0.09 |
| C031 | chr3:178952084 | C/T | C | PIK3CA | p.H1047Y | 0.06 |
| C031 | chr3:178952085 | A/G | A | PIK3CA | p.H1047R | 1.38 |
| C031 | chr12:25398281 | C/T | C | KRAS | p.G13D | 0.05 |
| C031 | chr12:25398284 | C/G | C | KRAS | p.G12R | 12.38 |
| C032 | chr3:178952085 | A/G | A | PIK3CA | p.H1047R | 1.15 |
| C032 | chr12:25398281 | C/T | C | KRAS | p.G13D | 0.27 |
| C032 | chr17:7578208 | T/C | T | TP53 | p.H214R | 0.06 |
| C033 | chr3:178952085 | A/G | A | PIK3CA | p.H1047R | 0.06 |
| C033 | chr7:140453136 | A/T | A | BRAF | p.V600E | 0.07 |
| C033 | chr12:25398284 | C/G | C | KRAS | p.G12R | 0.29 |
| C035 | chr3:178952072 | A/G | A | PIK3CA | p.M1043V | 0.15 |
| C035 | chr3:178952084 | C/T | C | PIK3CA | p.H1047Y | 0.09 |
| C035 | chr3:178952085 | A/G | A | PIK3CA | p.H1047R | 0.22 |
| C035 | chr7:140453136 | A/T | A | BRAF | p.V600E | 0.13 |
| C035 | chr12:25398280 | C/T | C | KRAS | p.G13D | 2.66 |
| C035 | chr12:25398284 | C/G | C | KRAS | p.G12R | 3.27 |
| C037 | chr3:178952085 | A/G | A | PIK3CA | p.H1047R | 0.1 |
| C037 | chr4:153249385 | G/A | G | FBXW7 | p.R465C | 0.08 |
| C037 | chr12:25398284 | C/G | C | KRAS | p.G12R | 0.19 |
| C037 | chr17:7577120 | C/T | C | TP53 | p.R273H | 0.06 |
| C037 | chr17:7577121 | G/A | G | TP53 | p.R273C | 0.38 |
| C037 | chr18:48575159 | C/T | C | SMAD4 | p.A118V | 0.3 |
| C040 | chr3:178952072 | A/G | A | PIK3CA | p.M1043V | 0.09 |
| C040 | chr3:178952085 | A/G | A | PIK3CA | p.H1047R | 0.13 |
| C040 | chr7:55227884 | C/T | C | EGFR | p.R451C | 0.11 |
| C040 | chr12:25398284 | C/G | C | KRAS | p.G12R | 0.48 |
| C040 | chr17:7578406 | C/T | C | TP53 | p.R175H | 56.86 |
| C040 | chr18:48575159 | C/T | C | SMAD4 | p.A118V | 0.07 |
| C041 | chr3:178952085 | A/G | A | PIK3CA | p.H1047R | 0.47 |
| C041 | chr12:25398281 | C/T | C | KRAS | p.G13D | 0.06 |
| C041 | chr12:25398284 | C/G | C | KRAS | p.G12R | 0.08 |
| C042 | chr17:7577547 | C/T | C | TP53 | p.G245D | 0.09 |
| C042 | chr17:7578461 | C/T | C | TP53 | p.V157F | 0.17 |
| C042 | chr17:7578475 | G/A | G | TP53 | p.P152L | 0.08 |
| C043 | chr3:178952085 | A/G | A | PIK3CA | p.H1047R | 0.06 |
| C043 | chr4:153245446 | G/A | G | FBXW7 | p.S582L | 7.31 |
| C043 | chr12:25398284 | C/G | C | KRAS | p.G12R | 0.11 |
| C043 | chr15:66774131 | G/A | G | MAP2K1 | p.E203K | 0.05 |
| C043 | chr17:7577539 | G/A | G | TP53 | p.R248W | 7.61 |
| C048 | chr3:178952085 | A/G | A | PIK3CA | p.H1047R | 0.27 |
| C048 | chr4:153249384 | C/T | C | FBXW7 | p.R465H | 5.95 |
| C048 | chr7:140453136 | A/T | A | BRAF | p.V600E | 0.24 |
| C048 | chr12:25398284 | C/G | C | KRAS | p.G12R | 11.37 |
| C048 | chr17:7577548 | C/T | C | TP53 | p.G245S | 5.34 |
| C049 | chr3:178952085 | A/G | A | PIK3CA | p.H1047R | 0.29 |
| C049 | chr12:25398284 | C/G | C | KRAS | p.G12R | 3.03 |
| C050 | chr3:178952085 | A/G | A | PIK3CA | p.H1047R | 0.25 |
| C050 | chr7:140453136 | A/T | A | BRAF | p.V600E | 0.09 |
| C050 | chr12:25380276 | T/C | T | KRAS | p.Q61L | 0.05 |
| C050 | chr12:25398284 | C/T | C | KRAS | p.G12D | 0.06 |
| C050 | chr17:7578271 | T/C | T | TP53 | p.H193R | 0.15 |
| C052 | chr3:178952085 | A/G | A | PIK3CA | p.H1047R | 0.09 |
| C052 | chr5:112174631 | C/T | C | APC | p.R1114Ter | 0.91 |
| C052 | chr12:25398284 | C/G | C | KRAS | p.G12R | 2.96 |
| C052 | chr17:7577557 | G/- | G | TP53 | p.S241F | 0.19 |
| C052 | chr20:57484420 | C/T | C | GNAS | p.R201C | 0.09 |
| C053 | chr3:178936082 | G/A | G | PIK3CA | p.E542K | 0.09 |
| C053 | chr3:178952085 | A/G | A | PIK3CA | p.H1047L | 0.25 |
| C053 | chr7:140453136 | A/T | A | BRAF | p.V600E | 0.36 |
| C053 | chr15:66727442 | T/A | T | MAP2K1 | p.F53C | 0.08 |
| C056 | chr3:178936091 | G/A | G | PIK3CA | p.E545K | 0.06 |
| C056 | chr4:153249384 | C/T | C | FBXW7 | p.R465H | 1.35 |
| C056 | chr12:25398281 | C/T | C | KRAS | p.G13D | 2.65 |
| C056 | chr12:25398284 | C/G | C | KRAS | p.G12R | 1.23 |
| C056 | chr18:48591918 | C/T | C | SMAD4 | p.R361C | 1.68 |
| C057 | chr5:112175639 | C/T | C | APC | p.R1450Ter | 0.07 |
| C057 | chr12:25398284 | C/G | C | KRAS | p.G12R | 2.52 |
| C057 | chr17:7577124 | C/A | C | TP53 | p.V272L | 0.06 |
| C057 | chr17:37880998 | G/A | G | ERBB2 | p.G776V | 0.44 |
| C058 | chr5:112175211 | AAAAG/- | AAAAG | APC | p.E1309fs | 0.1 |
| C058 | chr12:25398284 | C/A | C | KRAS | p.G12C | 0.09 |
| C058 | chr17:7577120 | C/T | C | TP53 | p.R273H | 0.07 |
| C058 | chr20:57484420 | C/T | C | GNAS | p.R201C | 0.06 |
| C058 | chr12:25398284 | C/G | C | KRAS | p.G12C | 0.1 |
| C059 | chr3:178952085 | A/G | A | PIK3CA | p.H1047R | 0.11 |
| C059 | chr12:25398284 | C/G | C | KRAS | p.G12R | 0.09 |
| C060 | chr12:25398284 | C/G | C | KRAS | p.G12R | 0.07 |
| C065 | chr12:25398284 | C/T | C | KRAS | p.G12S | 21.37 |
| C065 | chr20:57484420 | C/T | C | GNAS | p.R201C | 0.29 |
| C065 | chr20:57484421 | G/A | G | GNAS | p.R201H | 0.31 |
| C066 | chr3:178952085 | A/G | A | PIK3CA | p.H1047R | 2.09 |
| C066 | chr4:153249385 | G/A | G | FBXW7 | p.R465C | 0.06 |
| C066 | chr5:112173917 | C/T | C | APC | p.R876Ter | 2.23 |
| C066 | chr12:25398284 | C/T | C | KRAS | p.G12D | 2.15 |
| C067 | chr4:153245446 | G/A | G | FBXW7 | p.S582L | 0.08 |
| C068 | chr5:112175760 | TT/- | TT | APC | p.F1491fs | 0.15 |
| C068 | chr12:25398284 | C/G | C | KRAS | p.G12R | 0.13 |
| C072 | chr3:178952085 | A/G | A | PIK3CA | p.H1047R | 0.11 |
| C072 | chr12:25398284 | C/G | C | KRAS | p.G12R | 0.07 |
| C073 | chr17:7577538 | C/T | C | TP53 | p.R248Q | 9.5 |
| C073 | chr17:7577547 | C/T | C | TP53 | p.G245D | 0.05 |
| C073 | chr17:7577559 | G/A | G | TP53 | p.S241F | 0.05 |
| C074 | chr3:178936091 | G/A | G | PIK3CA | p.E545K | 4.24 |
| C074 | chr5:112173917 | C/T | C | APC | p.R876Ter | 3.23 |
| C074 | chr5:112175639 | C/T | C | APC | p.R1450Ter | 4.58 |
| C074 | chr12:25398284 | C/T | C | KRAS | p.G12D | 7.13 |
| C074 | chr18:48593406 | G/T | G | SMAD4 | p.G386D | 4.4 |
| C074 | chr20:57484421 | G/A | G | GNAS | p.R201H | 0.06 |
| C079 | chr3:178936091 | G/A | G | PIK3CA | p.E545K | 0.22 |
| C079 | chr20:57484420 | C/T | C | GNAS | p.R201C | 0.08 |
| C079 | chr20:57484421 | G/A | G | GNAS | p.R201H | 0.08 |
| C081 | chr12:25398284 | C/A | C | KRAS | p.G12V | 0.07 |
| C081 | chr17:7577120 | C/T | C | TP53 | p.R273H | 0.36 |
| C081 | chr17:7577538 | C/T | C | TP53 | p.R248Q | 0.06 |
| C088 | chr17:7577539 | G/A | G | TP53 | p.R248W | 83.42 |
| C102 | chr1:115256530 | G/T | G | NRAS | p.Q61K | 0.08 |
| C102 | chr7:140453136 | A/T | A | BRAF | p.V600E | 0.08 |
| C102 | chr17:7577509 | C/A | C | TP53 | p.E258K | 0.06 |
| C102 | chr17:7578508 | C/A | C | TP53 | p.C141Y | 0.06 |
| C102 | chr18:48591918 | C/A | C | SMAD4 | p.R361C | 0.06 |
| C102 | chr20:57484421 | G/A | G | GNAS | p.R201H | 0.09 |
| C106 | chr17:7577539 | G/A | G | TP53 | p.R248W | 0.06 |
| C106 | chr17:7578383 | G/- | G | TP53 | p.C182fs | 0.09 |
| C108 | chr5:112173704 | C/T | C | APC | p.R805Ter | 0.73 |
| C108 | chr5:112175207 | G/T | G | APC | p.E1306Ter | 1.01 |
| C108 | chr12:25398284 | C/A | C | KRAS | p.G12V | 0.69 |
| C108 | chr17:7577124 | C/T | C | TP53 | p.V272M | 0.08 |
| C112 | chr3:178952085 | A/G | A | PIK3CA | p.H1047R | 0.06 |
| C112 | chr5:112175639 | C/T | C | APC | p.R1450Ter | 1.48 |
| C112 | chr12:25398284 | C/T | C | KRAS | p.G12D | 0.65 |
| C112 | chr17:7577547 | C/G | C | TP53 | p.G245D | 0.92 |
| C114 | chr4:153249385 | G/A | G | FBXW7 | p.R465C | 0.24 |
| C114 | chr7:55227884 | C/T | C | EGFR | p.R451C | 0.08 |
| C114 | chr12:25398284 | C/T | C | KRAS | p.G12A | 0.25 |
